# Characteristics of women admitted to medium secure care: a comparison of patients admitted to specialised single-sex and mixed-sex services in an English forensic psychiatric hospital

**DOI:** 10.3389/fpsyt.2025.1712853

**Published:** 2025-12-11

**Authors:** Lucy McCarthy, Jodie Westhead, Simon Gibbon, Ruth M. Hatcher, Martin Clarke

**Affiliations:** 1 Nottinghamshire Healthcare NHS Foundation Trust, Leicester, United Kingdom; 2 National Confidential Inquiry into Suicide and Safety in Mental Health (NCISH), University of Manchester, Manchester, United Kingdom; 3 School of Psychology and Vision Sciences, University of Leicester, Leicester, United Kingdom

**Keywords:** admission, forensic, outcomes, psychiatry, single-sex, women

## Abstract

**Background:**

Forensic mental health care has evolved from mixed-sex provision to specialised sex-specific services. It is important to understand how the characteristics of women admitted to medium secure care have changed over time and how this may impact on their outcomes after discharge.

**Objective:**

The study aims to describe and compare admission and discharge characteristics of two consecutive cohorts; women admitted between 1983 and 2001 to a mixed-sex medium secure care (‘Mixed’ cohort) and women admitted between 2005 and 2013 to single-sex medium secure care (‘Specialised’ cohort).

**Methods:**

Data came from a 30-year study of outcomes for first admissions to an NHS medium secure hospital (the ALACRITy study). Follow-up data were available up to a census date of June 30^th^ 2013.

**Results:**

93 women comprised the Mixed cohort (mean age 29.3 years; 81% White ethnicity; 49% personality disorder diagnosis) and 45 women comprised the Specialised cohort (mean age 32.4 years; 76% White ethnicity; 49% personality disorder diagnosis). The Specialised cohort were more likely than the Mixed cohort to be admitted from high security, or under a forensic section of the Mental Health Act. The Specialised cohort were more likely than the Mixed cohort to have previous convictions, or to have committed a ‘grave’ index offence warranting a life sentence. Over 95% of all women had received previous inpatient psychiatric care. The Specialised cohort had greater prevalence of alcohol use, self-harm and childhood adversity than the Mixed cohort. At the census, 99% of the Mixed cohort and 42% of the Specialised cohort had been discharged. Women in the Specialised cohort had a longer median length of stay than the Mixed cohort; 859 days and 229 days respectively. Over 80% of patients in the Mixed cohort were readmitted during the follow-up period.

**Conclusion:**

The study provides empirical data for two consecutive cohorts of women admitted to one medium secure hospital over the course of thirty years. Women admitted to single-sex services had more criminological and adverse trauma histories than women admitted to the earlier mixed-sex service. Further research is required to establish the long-term outcomes of women admitted to specialised single-sex medium secure care.

## 1 Introduction

Secure psychiatric care operates at the interface of the criminal justice system and healthcare services, providing care and treatment to people with severe mental health problems who pose a significant risk to themselves or others (1). There are three levels of secure psychiatric care in England: High, Medium and Low. Prospective patients are assessed prior to admission to determine the most suitable level of care for their treatment and risk needs, however an inverse relationship exists between the number of psychiatric beds and the prison population (2). The landscape of secure psychiatric services in England has shifted over the last thirty years to focus on step-down care provided in the least restrictive environment. Between 2000 and 2018, the total number of beds in high secure care reduced from 1,290 (3) to 795 (4). An initial compensatory increase in capacity in English medium and low secure services, which were designed to “bridge the gap” between high secure and non-secure psychiatric care (5), mirrored the increasing demand for forensic mental health services in Europe and North America seen in the early 2000s (6).

During the redesign of the English secure care pathway, sex-specific policy considerations promoted the need for specialist services for women requiring secure psychiatric care (7, 8). Standard medium secure services for women were commissioned in response both to the growing reluctance amongst clinicians to admit women to mixed-sex wards (9) and consolidating evidence of how women’s characteristics and treatment needs differed from their male counterparts (10–12). A major driver for the development of single-sex services was a recognition that women patients in mixed-sex forensic services, many of whom have experienced physical or sexual abuse (13), are especially vulnerable to threats, harassment or abuse from male patients. Although it is noteworthy that a qualitative study investigating women’s experienced safety in single-sex and mixed-sex units reported that although women felt less vulnerable to sexual abuse, exploitation and physical assault within single-sex units, they still reported experiencing intimidation, threats and abuse from other women patients (14).

Following the reduction of English high secure beds for women from 345 in 1991, to 50 by 2008, Women’s Enhanced Medium Secure Services (WEMSS) were introduced, in an acknowledgement that existing medium secure provision may be unsuitable for some women previously residing high secure care (15). WEMSS operated with higher staff-to-patient ratios than standard medium secure services, and provided enhanced levels of relational and procedural security for women presenting with more complex therapeutic needs (15).

In 2013, women accounted for approximately 18% of the forensic inpatient population of England and Wales; this proportion is larger than many equivalent-sized European countries at that time (e.g. Italy 8%; Spain 8%) and double the average rate of 9.42% reported for twelve European countries examined by Tomlin and colleagues (16). Mapping exercises of the secure hospital estate in England have demonstrated both systematic reductions in the number of secure care beds for women over time (17–19) and distinct patterns of service provision, with most standard medium secure beds for women provided by the National Health Service (NHS) and low secure services for women predominantly provided by the independent sector (19). Contemporary service developments (e.g., New Models of Care/NHS-led provider collaboratives) have brought about further reductions in the medium and low secure bed estate for women, with their focus on reducing out of area placements, reducing transitions (through the introduction of blended medium/low services), and improving community-based provision (20, 21).

Medium secure services were originally considered to provide up to a 2-year inpatient stay (22). However, evidence indicates that admissions to medium secure care can be considerably greater than this, especially if patients present with complex needs (23, 24). In a national cohort study conducted in England and Wales, Maden and colleagues (25) reported an average length of stay of 259 days. More recently Ribiero and colleagues (26), reported a median length of stay of 465.5 days for the cohort of women also described by Tully et al. (27). Comparative international data for forensic patients’ length of stay is sparse and what is available shows considerable variability between countries (16). Nations’ legal frameworks, secure and non-secure healthcare provision and community resources all impact on the ability of services to move forensic patients along their care pathway (6) and preclude meaningful comparison of length of stay between nations.

Research has consistently demonstrated that women in secure care present with less criminality and more psychological distress than men (11, 12), and that they are less likely than men to have an index offence (IO) on admission (25, 28). When examining the characteristics of women residing in NHS and independent sector medium secure beds, Bartlett and colleagues (29) found that a quarter of patients had no recorded index offence and had been admitted under civil or informal sections of the Mental Health Act (MHA). Similarly, Tully and colleagues reported that 36% of women admitted to an NHS medium secure unit had no index offence (27).

Although women admitted to secure care are less likely than men to have previous convictions, particularly for more serious offences such as those of a violent or sexual nature (12, 30), they may be more likely than men to present with fire-setting behaviour (12). In their follow-up study of women admitted to an NHS medium secure unit, Tully and colleagues (27) reported that 16% of the women had an index offence of arson. Findings from 90 women admitted to an independent medium secure hospital between 2002 and 2010 (31) identified that 54% had a previous conviction for arson and/or a history of fire-setting behaviour without conviction. The variability seen in the literature around rates of offending or conviction for arson committed by women requires acknowledgement and may be partially explained by the observation that not all episodes of fire setting by women are pursued as offences and consequently do not result in a conviction. This is further illustrated by a previous study of patients with a history of arson admitted to medium secure hospital (32), which identified that 10 out of 36 women (all of whom are included within the cohorts of this current study) were noted to have set fires in the community or hospital but had not been convicted for these offences.

Women in custodial settings, including medium secure care frequently present with histories of childhood adversity and abuse (13, 25, 27). Adshead (33) reported that 81% of a sample of women referred to a forensic service had a history of childhood sexual abuse and 50% had a history of physical abuse. More contemporary studies of women referred to a medium secure service have identified that 51% had experienced sexual abuse, physical abuse or neglect (27). Women in secure care are also more likely to engage in self-harming behaviour than the general population (12, 34–38). A history of self-harm is a major predictor of self-harm incidents during admission to forensic services (39) however the prevalence of self-harm varies depending on the custodial setting (40). For example, Völlm and Dolan (37) report 45.9% of women prisoners had ever self-harmed or attempted suicide, and Bland and colleagues (13) report that 84% of women in one English high secure hospital had a recorded history of self- harm. In a meta-analysis that tested the potential for a direct relationship between childhood sexual abuse and self-harming behaviour in adulthood, Klonsky and Moyer (41) concluded that there was no causal relationship, and that the association between the two factors is due to correlation with other shared psychiatric risk factors such as family environment, alexithymia, hopelessness and personality disorder. Recent work by Holden and colleagues (42) provides more contemporary evidence for a cumulative influence of adverse childhood experiences (ACEs) increasing the likelihood of adult self-harm within a female forensic population. Holden and colleagues found that 81.9% or more of their sample had experienced one individual ACE (i.e., emotional abuse; physical abuse; sexual abuse; emotional neglect; physical neglect; parental separation or divorce; violence against mother; household alcohol/drug misuse; mental illness in household; or incarceration of household member). Moreover, 28.8% had experienced two to three ACEs and 37.9% had experienced four or more ACEs, and for every one-point increase in the number of ACEs, individuals were 1.62 times more likely to self-harm as adults (42).

Women admitted to medium secure care are more likely than men to have experienced psychiatric treatment in the community prior to admission (25, 27), or to be admitted from a high secure hospital (12). Whilst some studies have reported that women are less likely to have a history of alcohol and drug misuse than men (12), over a third of women in one high secure hospital had histories of substance misuse (38% alcohol; 37% drugs) (13) and other studies of women admitted to medium secure care report proportions of substance misuse of around 45% (27, 35).

Historically, research has suggested that the most common diagnosis amongst women admitted to medium secure care is personality disorder (12, 23, 25, 35). Contrary to this finding, Tully and colleagues (27) found that most women admitted to a London medium secure unit between 2008 and 2014 received a diagnosis of mental illness-specifically schizophrenia spectrum disorder. The same researchers found that the diagnosis of schizophrenia spectrum disorder was associated with ‘non-White’ ethnicity, whereas a diagnosis of personality disorder was associated with ‘White’ ethnicity (27). When examining differences between women admitted to NHS or independent provider services, Bartlett and colleagues (29) noted that NHS units typically treat women with major mental illness, reporting three times more women with a primary diagnosis of paranoid schizophrenia, with the independent sector focusing on women with personality difficulties. Bartlett et al. (29), also concluded that NHS medium secure services cater for women with a higher offending profile who have committed more serious offences as reflected by the higher use of criminal sections of the MHA.

Whilst research into the characteristics of women more recently admitted to services is sparse, literature examining their outcomes on discharge is also limited. Coid and colleagues (43) reported that one in seven (15%) of the 177 women discharged for medium secure psychiatric care across England were convicted of a criminal offence following discharge over a follow-up period of 6.2 years. Tully and colleagues (27) found that of the 40 patients for which hospital-held data were available, 17.5% reoffended following discharge and 26% of women were readmitted to a psychiatric hospital.

In this paper we provide comparative empirical data from one medium secure hospital (44), on the admission and discharge characteristics of women admitted before and after the introduction of specialist services for women. The paper focuses on an entire cohort of first admissions of women, over a thirty-year period, and extends our knowledge of admission and discharge characteristics of an under-studied patient group. As the landscape of secure care continues to evolve, and evidence-based practice builds (45), it is helpful to understand the clinical presentation, demographic characteristics and treatment needs of women historically admitted to medium secure care.

## 2 Method

### 2.1 Sample

The paper describes a cohort of 138 female patients, admitted to one English medium secure unit between July 1st 1983 and June 30th 2013, and utilises an anonymous subset of data from a larger longitudinal study of the outcomes of patients after their discharge from the unit (i.e., the ALACRITy study - please see Westhead et al. (46), and Davies et al. (47), for full methodological details). Ethical approval for the ALACRITy study was granted by the Health Research Authority Research Ethics Committee. The study was conducted under what is now, Section 251 of the NHS Act 2006 (48), and permitted data to be collected without participant consent. The project was also approved and sponsored by the NHS Trust of the unit where the study was conducted.

Patients were followed up from the point of discharge from their first admission to the unit, to the census date of June 30th 2013. We compared the characteristics of women admitted to either generic mixed-sex services (Mixed cohort; admissions from July 1983 to May 2001), or to single-sex services (Specialised cohort; admissions from November 2005 to June 2013). The Specialised cohort included patients admitted to either the standard Women’s Medium Secure care, or Women’s Enhanced Medium Secure Services (WEMSS). Under the ethical and legal framework of the ALACRITy study, it was not possible to disaggregate data from these two services. For clarity, all patients included in the study were biologically female at birth.

### 2.2 Data collection and analysis

ALACRITy data were obtained with the requisite permissions from hospital records, the Offenders Index, Police National Computer (Ministry of Justice) and NHS Digital. This paper reports on a number of admission and discharge variables for which there was minimal missing data in both cohorts. Cases were categorized according to the 1983 Mental Health Act (MHA) (49) of Mental Illness and Psychopathic Disorder; as Arnold Lodge does not provide services for people with Mental Impairment and Severe Mental Impairment, these two classifications did not apply. Patients categorised under the MHA classification of Mental Illness experienced severe mental illness such as schizophrenia, bipolar disorder or psychotic depression. Patients categorised under the MHS classification of Psychopathic Disorder experienced personality disorder often comorbid with non-psychotic mental illnesses.

Categorical and continuous admission variables described patients’ Demographics, Childhood and Family history, Criminal history, and Psychiatric history at admission to the unit. Categorical and continuous discharge variables described patients’ course after discharge from the unit.

Categorical admission variables: (Demographics: Ethnic identity; Forensic MHA Section; MHA classification; International Classification of Diseases 10 (50) Diagnosis on admission); (Childhood and Family history: Child physical abuse; Child sexual abuse; Intra-familiar abuse; Extra-familiar abuse; Parental Separation before the age of 18 years; History of non-secure institutional care; Childhood problematic behaviours; Childhood aggression and violence; Contact with child psychiatrist/psychologist); (Criminal history: Index Offence (IO) on admission; IO offence type; Previous acquisitive offending; Previous violent offending; Custody as adult; Juvenile sentences); (Psychiatric history: Non-secure and Secure psychiatric admissions; Prior self-harm; Self-harm during admission; Alcohol use; Drug use). Continuous admission variables: (Demographics: Age on admission); (Criminal history: Age at first conviction; Number of previous convictions).

Categorical discharge variables: Discharged from the unit; Discharge location; Readmission to hospital (defined as readmission to non-secure and secure psychiatric services from prison, the community, or a lower level of secure care); Reconviction during follow-up. Continuous discharge variables: ‘Time at risk’ (defined as the length of time from discharge to date of death or census); Length of stay at the unit; Time to first re-admission; Time to reconviction.

Data were analysed using Jamovi (v2.6.24). Frequencies and descriptive statistics (mean, standard deviation) were reported; where data were skewed, median values were reported. Continuous variables were compared using parametric Students t-tests, or non-parametric tests (Mann-Whitney U) where the Gaussian assumptions of normality were not met; 95% confidence intervals, and effect sizes were presented. Categorical data were compared using Chi-square tests, with effect sizes presented as odds ratios (OR) with 95% confidence intervals. Statistical disclosure control was used throughout when reporting variables with counts of five or under.

## 3 Results

### 3.1 Comparison of baseline admission characteristics

Ninety-three women were admitted to mixed-sex services at a Medium Secure Unit (MSU), over a 214-month period (the Mixed cohort). A further 45 women were admitted to the same MSU over a 91-month period, to two single-sex services (the Specialised cohort). The primary baseline demographic admission characteristics of the two cohorts are provided in **Table 1**.

**Table 1:** Admission characteristics.

Categorical variables	Mixed services n (%)	Specialised women’s services n (%)	Odds ratio	*X*^2^ (df)	p
Ethnic identity of White British/Irish (n=138)	75 (80.65)	34 (75.56)	0.74 (0.32 to 1.74)	0.47 (1)	0.491
Admitted on a Forensic MHA Section (n=136) (2 informal patients in Mixed cohort excluded)	53 (58.24)	37 (82.22)	3.32 (1.39 to 7.92)	7.74 (1)	0.005
MHA class of Psychopathic Disorder (n=136) (2 informal patients in Mixed cohort excluded)	40 (43.96)	23 (51.11)	1.33 (0.65 to 2.73)	0.62 (1)	0.431
ICD Diagnosis on admission (NB missing data, n=42 for Mixed services cohort) (n=96)	N=51	N=45	N/A	3.14 (3)	0.370
F20-29: Schizophrenia, Schizotypal & Delusional disorders	20 (39.22)	15 (33.33)	.	.	.
F30-39: Mood disorders	3 (5.88)	7 (15.56)	.	.	.
F60-69: Disorders of adult personality and behaviour	25 (49.02)	22 (48.89)	.	.	.
Other: F40-48, F296, F300	3 (5.88)	1 (2.22)	.	.	.
Index Offence (IO) on admission (Yes) (n=138)	61 (65.59)	39 (86.67)	3.41 (1.31 to 8.91)	6.75 (1)	0.009
Grave IO (n=100)	19 (31.15)	20 (51.28)	0.43 (0.19 to 0.99)	4.05 (1)	0.044
Murder/Manslaughter IO (n=100)	<5 (<10%)	5 (<10%)	1.61 (0.31 to 8.42)	0.33 (1)	0.569
Attempted murder, GBH, Section 18 wounding IO (n=100)	9 (14.75)	7 (17.95)	1.26 (0.43 to 3.73)	0.18 (1)	0.671
Assault/ABH IO (n=100)	7 (11.48)	6 (15.38)	1.40 (0.43 to 4.53)	0.32 (1)	0.571
Simple arson IO (n=100)	21 (34.43)	5 (12.82)	0.28 (0.10 to 0.822)	5.77 (1)	0.016
Arson with intent IO (n=100)	7 (11.48)	8 (20.51)	0.50 (0.17 to 1.52)	1.52 (1)	0.217
History of child physical abuse (n=126)	29 (35.80)	28 (62.22)	2.95 (1.39 to 6.28)	8.15 (1)	0.004
History of sexual abuse (n=125)	45 (56.25)	35 (77.78)	2.72 (1.19 to 6.24)	5.79 (1)	0.016
Intra-familiar sexual abuse (n=124)	29 (36.71)	22 (48.89)	1.62 (0.79 to 3.46)	1.76 (1)	0.185
Extra-familiar sexual abuse (n=124)	27 (33.75)	21 (47.73)	1.79 (0.85 to 3.80)	2.34 (1)	0.126
Parental separation before age 18 (n=132)	39 (44.32)	23 (52.27)	0.73 (0.35 to 1.50)	0.75 (1)	0.388
History of non-secure institutional care (n=132)	28 (32.18)	24 (53.33)	0.42 (0.20 to 0.87)	5.56 (1)	0.018
History of childhood problematic behaviours (e.g., truanting or stealing) (n=120)	46 (56.79)	26 (66.67)	1.52 (0.69 to 3.38)	1.07 (1)	0.301
History of childhood aggression and violence (n=123)	30 (37.04)	28 (66.67)	3.40 (1.55 to 7.45)	9.74 (1)	0.002
Contact with child psychologist/psychiatrist (n=133)	36 (40.91)	32 (71.11)	3.56 (1.64 to 7.70)	10.87 (1)	0.001
Juvenile sentences (n=135)	7 (7.78)	5 (11.11)	1.48 (0.44 to 4.96)	0.41 (1)	0.521
Previous Inpatient (n=136)	88 (96.70)	44 (97.78)	1.50 (0.15 to 14.80)	0.12 (1)	0.727
Previous High secure admission (n=135)	12 (13.33)	11 (24.44)	2.10 (0.85 to 5.23)	2.62 (1)	0.106
Previous Medium secure admission (n=134)	5 (5.62)	24 (53.33)	19.19 (6.55 to 56.29)	40.13 (1)	<0.001
Previous Non-secure admission (n=136)	86 (94.51)	36 (80.00)	4.30 (1.35 to 13.70)	6.86 (1)	0.009
Custody as adult (n=135)	18 (20.00)	22 (48.89)	3.38 (1.75 to 8.35)	12.01 (1)	<0.001
Previous acquisitive offence (including IO)	31 (33.33)	19 (42.22)	0.68 (0.33 to 1.42)	1.04 (1)	0.308
Previous violent offence (including IO) (i.e., Homicide, Wounding, GBH, Robbery, ABH, Assault, Other violent offence) (n=138)	45 (48.39)	28 (62.22)	0.57 (0.28 to 1.18)	2.33 (1)	0.127
Previous alcohol use (Yes) (n=117)	21 (28.37)	34 (79.10)	9.53 (3.91 to 23.30)	28.05 (1)	<0.001
Previous drug use (Yes) (n=111) *(Excludes missing data n=27 in Mixed cohort)*	58 (87.88)	37 (82.22)	1.57 (0.54 to 4.54)	0.69 (1)	0.405
Previous self-harm (Yes) (n=135)	65 (72.22)	43 (95.56)	8.27 (1.86 to 36.70)	10.21 (1)	0.001
Life threatening self-harm during admission (Yes) (n=134)	71 (79.79)	40 (88.89)	2.03 (0.70 to 5.88)	1.75 (1)	0.186

Continuous variables	Mixed services	Specialised women’s services	Statistic	df	p
Age on admission (years) / Mean (SD) (n=138)	29.3 (8.9)	32.4 (9.0)	t= -1.89, md= -3.07, (95% CI -6.28 to 0.15)	136	0.061
Age at first conviction (years) / Mean (SD) (n=97)	20.7 (8.2)	22.1 (9.2)	t= -0.77, md= -1.37, (95% CI -4.93 to 2.18)	95	0.446
Number of previous convictions / Median (IQR) (n=135)	1.0 (3.00)	2.0 (7.00)	U=1553, Z= -2.01	.	0.037

#### 3.1.1 Age and ethnicity

The mean age at admission was similar in both cohorts (29.3 years for the Mixed cohort versus (vs.) 32.4 years for the Specialised cohort, t=-1.89, df=136, p=0.061).

Women in the Mixed, and Specialised cohorts were similar in their ethnic background with more than three quarters in either group recorded as white British/Irish (80.65% of Mixed; 75.56% of Specialised), compared with any other ethnic background (19.35% of Mixed; 24.44% of Specialised).

#### 3.1.2 MHA classification

A higher proportion of women in the Specialised cohort were admitted under a forensic Mental Health Act (MHA) section, as opposed to a civil MHA section (82.22% vs., 58.24%, p=0.005). However, the proportion of women detained under a MHA classification of Psychopathic Disorder (akin to personality disorder), compared with any other MHA Classification, was similar in the two cohorts (X^2^ = 0.620 (1df), p=0.431).

#### 3.1.3 Diagnosis on admission

Extensive missing data was observed in the Mixed cohort for the coding of diagnosis on admission. From the available data, similar proportions of patients in the Mixed and Specialised cohorts had a primary diagnosis of psychotic or delusional illnesses defined by ICD-10 (50) F20-29 (39.22% vs., 33.33%), and disorders of adult personality and behaviour (F60-69, F301) (49.02% vs., 48.89%). A small proportion of patients in both cohorts were admitted with a diagnosis of mood disorder (F30-39) (5.88% for Mixed and 15.56% for Specialised), or other diagnoses (5.88% for Mixed, 2.22% for Specialised).

#### 3.1.4 Source of admission

Compared to the Mixed cohort, a higher proportion of women in the Specialised cohort were admitted from High security [admitted yes/no comparison] (20.0% vs., 7.53%, X^2^ = 4.603 (1df), p=0.032), or Non-NHS Secure services (57.78% vs., 1.08%, X^2^ = 61.957 (1df), p<0.001). A smaller proportion of women in the Specialised cohort were admitted from Prison/Remand compared with the Mixed cohort (22.22% vs., 43.01%, X^2^ = 5.67 (1df), p=0.017).

#### 3.1.5 Criminal history, including index offence

A higher proportion of women in the Specialised cohort had an index offence compared to the Mixed cohort (86.67% vs., 65.59%, p=0.009). Of those with an index offence on admission (n=100), more women in the Specialised cohort committed a ‘grave’ index offence (i.e. an offence warranting a life sentence), as opposed to a standard offence, than those the Mixed cohort (51.28% vs., 31.15%, p=0.044). There was no difference between the Specialised and Mixed cohorts in the proportion of women with a primary index offence of murder/manslaughter (<10% vs., <10%, p=0.569), attempted murder/Grievous Bodily Harm (GBH)/Section 18 wounding (17.95% vs., 14.75%, p=0.671), assault/actual bodily harm (ABH) (15.38% vs., 11.48%, p=0.571) or arson with intent to endanger life (or reckless as to whether life endangered) (20.51% vs., 11.48%, p=0.217). A smaller proportion of women in the Specialised cohort than the Mixed cohort were noted to have a primary index offence of simple arson (i.e., arson without intent to endanger life) (12.82% vs., 34.43%, p=0.016).

Women in the Specialised cohort had a larger number of previous convictions than those in the Mixed cohort (Median (IQR) 2.00 (7.00) vs., 1.00 (3.00), p=0.037), and a larger proportion of women in the Specialised cohort had previously received a custodial sentence as an adult compared to the Mixed cohort (48.89% vs., 20.00%, p<0.001). However, there was no difference in the proportion of women in the Specialised and Mixed cohorts with a prior conviction for an acquisitive offence (42.22% vs., 33.33%, p=0.308), or a violent offence (i.e., Homicide, Wounding, GBH, Robbery, ABH, Assault, Other violent offence) (62.22% vs., 48.39%, p=0.127). There was no difference in the age of first conviction for women in Specialised and Mixed cohorts (Mean (SD) 22.1 (9.2) years vs., 20.7 (8.2) years, p=0.446), and there was no difference in the proportion having a juvenile sentence (11.11% vs., 7.78%, p=0.521).

#### 3.1.6 Previous psychiatric history

Over 95% of all the women had previously received inpatient psychiatric care prior to admission to the unit, with a larger proportion of women in the Mixed cohort experiencing a non-secure psychiatric admission, compared to those in the Specialised cohort (94.51% vs., 80.00%, p=0.009). A larger proportion of women in the Specialised cohort had experienced a previous admission to medium secure care (53.33% vs., 5.62%, p<0.001), but there was no statistical difference in the proportion of women with a previous admission to high secure care (24.44% vs., 13.33%, p=0.106).

#### 3.1.7 Self-harm

The prevalence of previous self-harm was high generally (>70%), with a larger proportion of women in the Specialised cohort than the Mixed cohort (95.56% vs., 72.22%, p=0.001). A very high proportion of patients in both cohorts (>80%) exhibited life-threatening self-harm behaviour during their admission.

#### 3.1.8 Alcohol and substance misuse

Problematic alcohol use was identified in a larger proportion of women in the Specialised cohort, compared with the Mixed cohort (79.10% vs., 28.37%, p<0.001), and although there was a high rate of drug use reported, there was not a statistical difference between the two cohorts (82.22% vs., 87.88%, p=0.405).

#### 3.1.9 Childhood characteristics

As can be seen in **Table 1**, a greater proportion of women in the Specialised cohort experienced childhood abuse and adversity than women in the Mixed cohort. Statistically significant differences were identified in the history of child physical abuse (62.22% vs., 35.80%, p=0.004) and history of sexual abuse (77.78% vs., 56.25%, p=0.016), however there was no difference in the proportion of each cohort reporting intra-familiar sexual abuse (48.89%, vs., 36.71%, p=0.185), or extra-familiar sexual abuse (47.73% vs., 33.75%, p=0.126).

There was no difference between the cohorts in the experience of parental separation before the age of 18 years (52.27% vs., 44.32%, p=0.388), however a larger proportion of women in the Specialised cohort had a history of non-secure institutional care (i.e., being ‘looked after’ in a children’s home) than in the Mixed cohort (53.33% vs., 32.18%, p=0.018).

Over half of the all the women displayed problematic behaviours in childhood (such as truanting or stealing) and although this did not differ significantly between the two cohorts (66.67% vs., 56.79%, p=0.301), a greater proportion of women in the Specialised cohort were identified as having a history of childhood aggression/violence than those in the Mixed cohort (66.67% vs., 37.04%, p=0.002). A larger proportion of the Specialised cohort had contact with a child psychologist or psychiatrist, than the Mixed cohort (71.11% vs., 40.91%, p=0.001).

### 3.2 Comparison of discharge characteristics

Discharge outcome data for the two cohorts is presented in **Table 2** with the caveat that as the Mixed cohort pre-dates the Specialised cohort, the time available for follow-up differed markedly between the groups. The proportion of patients who had been discharged from the unit was significantly different between the cohorts (Mixed 98.92% vs., Specialised 42.22%, p=0.001). The length of time (in months) spent ‘at risk’ during the follow-up period was also statistically different between the two cohorts (Mixed cohort median time at risk = 242 months; Specialised cohort = 34.4 months, Mann Whitney U = 54.0, p<0.001). Descriptive statistics for post-discharge readmission and reconviction variables are provided, however, as the data would be impacted by differences in exposure or time ‘at risk’, the statistical test of differences between cohorts is not reported.

**Table 2:** Discharge variables.

Categorical variables	Mixed services n (%)	Specialised women's services n (%)	Odds ratio	*X*^2^ (df)	p
Discharged from the unit (i.e., had time in the community/prison/other hospital during follow-up period) (Yes) (n=138)	92 (98.92)	19 (42.22)	126 (16.1 to 985.0)	62.0 (1)	<0.001
Discharge location (n=118) 20 women in Specialised cohort not discharged at census date.	n=93	n=25	N/A	Not reported	
Community	26 (27.96)	<5 (<10)	.	.	.
Prison	9 (9.68)	<5 (<10)	.	.	.
High secure hospital	14 (15.05)	<5 (<20)	.	.	.
Medium secure hospital	<5 (<10)	<5 (<10)	.	.	.
Low secure hospital	<5 (<10)	14 (56.00)	.	.	.
General non-secure psychiatric hospital including PICU	39 (41.94)	<5 (<10)	.	.	.
Other	<5 (<10)	<5 (<10)	.	.	.
Security level of discharge location, relative to admission source (n=118)	n=93	n=25	N/A	9.01 (2)	0.011
Higher	23 (24.73)	3 (12.00)	.	.	.
Neutral	41 (44.09)	6 (24.00)	.	.	.
Lower	29 (31.18)	16 (64.00)	.	.	.
Re-admission (n=116)	n=91	n=25	.	.	.
Not re-admitted	16 (17.58)	16 (64.00)	NA	Not reported	
Re-admitted to non-secure care only	28 (30.77)	<5 (<20)	.	.	.
Re-admitted to secure care only	18 (19.78)	<5 (<20)	.	.	.
Readmitted to both non-secure and secure care	29 (31.87)	<5 (<10)	.	.	.
Reconviction during follow-up (Yes) (n=118)	38 (40.86)	<5 (<10)	NA	Not reported	.

Continuous variables	Mixed services	Specialised women's services	Statistic	-	p
Time ‘at risk’ during follow-up (months) / Median (IQR) (n=118)	242 (75.7)	34.4 (24.3)	U=54.0, Z=-7.30	.	<0.001
Length of stay (days) / Median (IQR) (n=118)	229 (355)	859 (593)	U=382, Z= -5.14	.	<0.001
Time to first re-admission (years) / Median (IQR) (n=66)	1.66 (4.51)	1.69 (0.66)	U=146, Z=-0.16	.	0.884
Time to reconviction (years) / Median (IQR) (n=40)	1.81 (5.15)	2.12 (0.58)	NA Not reported	.	.

The median length of stay for women in the Specialised cohort was significantly longer than that of those in the Mixed cohort (859 days vs., 229 days, p<0.001). The low number of discharges to specific locations preclude statistical analysis of differences between the cohorts, but the distribution of discharges appears to differ between the two cohorts. Women in the Mixed cohort were often discharged to a general non-secure psychiatric hospital (41.94%) or to the community (27.96%); Patients in the Specialised cohort were often discharged to low secure hospital (56.00%). A similar proportion of women were discharged to high secure hospital (15.05% Mixed, <20% Specialised). There was a significant difference in the security level of the discharge location, relative to the admission source, between the proportions of women in the two cohorts (X^2^ = 9.01 (2df), p=0.011). Proportionally, more women in the Mixed cohort than the Specialised cohort were discharged to a location that was the same level of security as their admission sources (44.09% vs., 24.00%), or to a higher level of security (24.73% vs., 12.00%). Conversely, a higher proportion of women in the Specialised cohort than the Mixed were discharged to a location that was of a lower security than their admission source (64.00% vs., 31.18%).

#### 3.2.1 Readmission

A total of 116 patients from the two cohorts had time ‘at risk’ for readmission following their discharge from the MSU. There was no difference in the median length of time to re-admission to any psychiatric hospital, between the two cohorts (1.66 years for the Mixed, 1.69 years for the Specialised, p=0.884).

Over eighty percent of women in the Mixed cohort were re-admitted during the follow-up; 30.77% to non-secure care, 19.78% to secure care, and 31.87% to both. A small number of women from the Specialised cohort were readmitted to non-secure care, secure care, or both.

#### 3.2.2 Reconviction

Forty percent (40.86%) of the Mixed cohort and less than 10% of the Specialised cohort were convicted of an offence committed after discharge from the unit. The disparity of time in follow-up between the Mixed and Specialised cohorts, and the small number of discharges in the Specialised cohort, makes statistical comparison inappropriate.

## 4 Discussion

### 4.1 Main findings

This paper provides empirical data for two consecutive cohorts of women admitted to one English medium secure hospital. The basic demographics (i.e., age on admission; ethnicity; MHA classification on admission) of the two cohorts were similar, with a large proportion of patients with white British/Irish ethnic identity and/or a MHA classification of psychopathic disorder. However, there were important differences in the admission characteristics of the two cohorts.

The Specialised cohort had more significant criminological backgrounds than the Mixed cohort. The women in the Specialised cohort were more likely to have an index offence (especially a more serious or ‘grave’ index offence), have a larger number of previous convictions, experienced custody as an adult, and unsurprisingly were more likely to be admitted under a forensic MHA section. The increased criminality observed in the Specialised cohort may be partially related to the fact that more of these patients were admitted as part of a step down from high secure care, than patients in the Mixed cohort. It is also possible (and concordant with the Penrose hypothesis (2)), that the increase in the prison population, coupled with the overall reduction in secure psychiatric beds, resulted in patients with increasingly severe clinical acuity being admitted from prison over the course of the study period.

An index offence of arson was the most common individual offence in both cohorts, with the more serious offence of arson ‘with intent’ more prevalent in the Specialised cohort and simple arson (i.e., arson ‘without intent’) more prevalent in the Mixed cohort. Although most of the Mixed cohort had experienced non-secure psychiatric admissions before their admission to the unit, women in the Specialised cohort were more likely to have had a previous medium secure admission (especially in non-NHS hospitals), or to be admitted to the unit from high secure hospital, as would be expected given the inclusion of WEMSS patients within the Specialised cohort.

Adverse childhood experiences and their sequalae (such as physical and sexual abuse, institutional care, childhood aggression/violence, and contact with child psychiatrist/psychologist) were observed more frequently in the Specialised cohort. Although this observation may be an artifact of better recording practices and improved awareness of the salience of childhood adversities in the development of adult psychiatric disorders, the higher levels of alcohol abuse and self-harm also seen in the Specialised cohort, may reflect adulthood responses to experiences of childhood trauma observed in this group.

The average length of stay was significantly longer for the Specialised cohort than the Mixed cohort. This may reflect the interaction between high clinical acuity and more complex criminological treatment needs of the women directed to sex-specific services.

The observed differences in the clinical acuity of patients in the two cohorts, coupled with changes in the number of secure mental health beds commissioned over the course of the ALACRITy study, make it difficult to assess the significance of differences in the discharge locations of the two cohorts. Despite a reduction in the English high secure estate, a small, and similar proportion of patients in both cohorts were discharged up to high secure hospital. Most patients in the Specialised cohort were discharged to low secure care, whereas most of the Mixed cohort were discharged to non-secure psychiatric hospital, or the community; this finding reflects the introduction of low secure services to the forensic treatment pathways. Compared with the security-level of the admission source, a larger proportion of patients in the Specialised cohort were discharged to a lower level of security than the Mixed cohort.

The length of follow-up was significantly different for the two groups; almost all the Mixed cohort spent time out of hospital or prison during the follow-up period, compared with just over 40% of the Specialised group. Due to this confounding factor, we were unable to meaningfully comment on differences in post-discharge readmission or reconviction between the two cohorts. Nonetheless the observation that most of the Mixed cohort were re-admitted to psychiatric care during the follow-up period is important to note, especially as the Mixed cohort presented with less historic trauma and complexity than the Specialised cohort.

### 4.2 Comparison with other studies

By collating evidence from the diagnosis on admission data (for which there was missing data in the Mixed cohort) and the proportion of patients with a MHA Classification of Psychopathic disorder, we can be confident that personality disorder was the most frequent diagnosis on admission - accounting for approximately half of all diagnoses, and corresponds with the findings of previous studies (12, 23, 25, 31). As the ethnicity of both cohorts was predominately White, these results align with the findings of Tully and colleagues (27) who observed a strong association between diagnosis of personality disorder and White ethnicity. Given the multicultural population that the unit serves, it is unclear whether there is unmet need for forensic care within populations of patients from the global majority, especially those with major mental illness.

The proportion of women without an index offence differed between the cohorts, with the Mixed cohort incidence of 34%, comparing favourably to the 25% reported by Bartlett et al. (29), and being similar the 36% reported by Tully et al. (27). In comparison, only 13% of the Specialised cohort were without an index offence on admission. For those with an index offence, the offence of arson (with or without intent) was observed in nearly a half of the Mixed cohort (46%), and a third of the Specialised cohort (33%); these figures fall between the reported levels of arson (16% and 54%) observed by Tully et al. (27), and Long et al. (31), respectively.

Women in both cohorts reported significant levels of childhood adversity and self-harm, supporting the previous observations of Maden and others (13, 25, 27, 33). Rates of historical self-harm were high (>70%) in both cohorts, as were rates of life-threatening self-harm during admission (>80%); these results align with the findings of Kaggwa and colleagues who reported that a history of self-harm is seen to be a strong predictor of future self-harm behaviour among forensic patients (39).

Three study variables (child physical abuse, child sexual abuse, and parental separation) were comparable to ACEs reported by Holden and colleagues (42). A larger proportion of patients in both the Mixed and Specialised cohorts had histories of these ACEs, than that reported by Holden (e.g. Child physical abuse: 36% Mixed cohort, 62% Specialised cohort, vs., 41% Holden sample; Child sexual abuse: Mixed cohort 56%, Specialised cohort 78%, vs., 30.0% Holden sample; Parental separation: 44% Mixed cohort, 52% Specialised cohort, vs., 39.4% Holden sample).

The increased prevalence of childhood abuse, trauma, substance use, previous secure psychiatric admissions and self-harm seen in the Specialised cohort, aligns with the increased therapeutic needs of women requiring enhanced medium secure care described by Sarkar and di Lustro (15).

### 4.3 Strengths and limitations of the study

The study provides informative empirical data for a population of women admitted to a medium secure hospital over a thirty-year period. The long study timeframe inevitably means that there will have been changes in national and regional service provision, legislative frameworks and population characteristics that may influence individual patient outcomes. Nonetheless we maintain that for the women admitted to the service, the most impactful variation during this time would be of being admitted to either a mixed-sex, or a single-sex service.

The study highlights the multiple vulnerabilities of women admitted to forensic services, regardless of whether they are admitted from community services, or as a step-down from a high secure hospital. The authors acknowledge however, that the single-site, retrospective nature of the ALACRITy study, and the passage of time from the end of outcome data collection to the present day, reduces the potential relevance and comparability of this data to contemporary patient cohorts. Improvements in clinical assessment and recording since the 1980s (e.g., in recording of adverse childhood experiences, alcohol and substance use, or diagnostic coding), may account for the more complete data records in the Specialised cohort, but the temporal difference in the two cohorts remains the most significant limitation to the study. Additionally, were the study to be conducted today, we might reasonably be expected to consider additional clinical characteristics (such as neurodiversity or complex post-traumatic stress disorder) that are not included in our data. As the Mixed cohort and Specialised cohort represent different time periods and treatment philosophies, they also have different lengths of follow-up time and ‘time at risk’ in the community. We have reported basic post-discharge outcomes, for the sake of transparency, but we are unable to make strong comparisons between the cohorts or draw conclusions on how well each group of women has fared after discharge. Future research might be undertaken to follow-up the Specialised cohort for an additional period of time, to allow such comparisons to be made.

An additional major limitation of the study is the conflation of the standard women’s and enhanced women’s secure services (WEMSS) into one Specialised women’s cohort. Whilst both services offer sex-specific care, there are differences in both the characteristics of women admitted to the WEMSS and in the treatment offered within the enhanced service. Data governance arrangements for the use of ALACRITy study data preclude identification of women admitted to the standard and enhanced women’s services.

### 4.4 Potential future research

Whilst recognising the limitations of this study, we also acknowledge that this paper pragmatically provides empirical data for a historically under-researched population. Our findings highlight a number of areas where further research would be particularly helpful.

Firstly, a better understanding of the relationships between ethnicity, diagnosis, and referral to secure care for women, would help commissioners identify potential unmet clinical need, and assist clinicians to deliver specialist care to all patients that might benefit.

Secondly, it would be helpful to have comparative follow-up studies focused on the treatment provision, length of stay and outcomes of women admitted to standard and enhanced medium secure services. As commissioning arrangements for women’s secure beds continue to evolve, understanding the evidence base around successful outcomes is essential for the benefit of the vulnerable women who require these services.

A third area for future research might examine the prevalence and impact of neurodiversity within the women’s medium secure estate. The historical and retrospective nature of the ALACRITy study data precluded any systematic comparison of neurodivergence between the two cohorts, as data were not routinely available. Anecdotal evidence suggests that contemporary services are becoming more attuned to recognising and identifying the clinical needs of women presenting with neurodivergence (in addition to the other vulnerabilities seen in forensic populations). It will be important to assess the long-term outcomes of this clinically distinct population.

A number of variables reported in this paper (i.e., child sexual abuse, child physical abuse, parental separation) are acknowledged ACEs. Additional variables collected for the ALACRITy study, but not reported here, may allow researchers to infer additional ACEs (such as family history of mental illness, incarceration of household member, or household alcohol/drug misuse). Future research might look at the prevalence of ACEs in the two cohorts and relate this to the long-term outcomes of the ALACRITy study (i.e., readmission, reoffending and mortality).

### 4.5 In conclusion

This study provides ‘real world’ empirical follow-up data for two consecutive cohorts of women admitted to one English medium secure hospital. The study identifies demographic differences between women admitted to mixed-sex services between 1983 and 2001 and those admitted to single-sex services between 2005 and 2013. Women admitted to the Specialised services generally had more criminological and adverse/trauma histories than women admitted to Mixed-sex services. The consecutive nature of the cohorts is a major limitation to the study, nonetheless the study provides clinicians and commissioners with novel evidence regarding the admission characteristics, treatment pathways, and outcomes of women admitted to medium secure hospital.

## Data Availability

The datasets presented in this article are not readily available due to confidentiality. Requests to access the datasets should be directed to lucy.mccarthy@nottshc.nhs.uk.

## References

[1] Boyd-Caine T . Protecting the public? Detention and release of mentally disordered offenders. Oxford: Routledge (2012). doi: 10.4324/9780203833315

[2] Wild G Alder R Weich S McKinnon I Keown P . The Penrose hypothesis in the second half of the 20th century: Investigating the relationship between psychiatric bed numbers and the prison population in England between 1960 and 2018–2019. Br J Psychiatry. (2022) 220:295–301. doi: 10.1192/bjp.2021.138, PMID: 35049470

[3] Fender A . Modelling Strategic Changes in High Secure Hospital Services. London: High Secure Psychiatric Services Commissioning National Oversight Group (2000).

[4] Duke L Furtado V Guo B Völlm BA . Long-stay in forensic psychiatric care in the UK. Soc Psychiatry Psychiatr Epidemiol. (2018) 53:313–21. doi: 10.1007/s00127-017-1473-y, PMID: 29387921 PMC5842247

[5] Home Office & Department of Health and Social Security . Report of the Committee on Mentally Abnormal Offenders (The Butler Report; Cmnd 6244) (1975). London: HMSO. Available online at: https://wellcomecollection.org/works/steazp4r/items (Accessed November 25, 2025).

[6] Jansman-Hart EM Seto MC Crocker AG Nicholls TL Côté G . International trends in demand for forensic mental health services. Int J Forensic Ment Health. (2011) 10:326–36. doi: 10.1080/14999013.2011.625591

[7] Department of Health . Women's Mental Health: Into the Mainstream, Strategic Development of Mental Health Care for Women (2002). London: Department of Health. Available online at: https://data.parliament.uk/DepositedPapers/Files/DEP2009-0974/DEP2009-0974.pdf (Accessed November 25, 2025).

[8] Department of Health . Mainstreaming Gender and Women's Mental Health: Implementation Guidance (2003). London: Department of Health. Available online at: https://archive.org/details/b32231945/page/14/mode/2up (Accessed November 25, 2025).

[9] Ricketts D Carnall H Davies S Kaul A Duggan C . First admissions to a regional secure unit over a 16-year period: changes in demographic and service characteristics. J Forensic Psychiatry. (2001) 12:78–89. doi: 10.1080/09585180121913

[10] Thomas SDM Dolan M Shaw J Thomas S Thornicroft G Leese M . Redeveloping secure psychiatric services for women. Medicine Sci Law. (2005) 45:331–9. doi: 10.1258/rsmmsl.45.4.331, PMID: 16302379

[11] Lart R Payne S Beaumont B Macdonald G Mistry T . Women and Secure Psychiatric Services: A literature Review (1999). York: NHS Centre for Reviews and Dissemination. Available online at: https://www.york.ac.uk/media/crd/crdreport14.pdf (Accessed November 25, 2025).

[12] Coid J Kahtan N Gault S Jarman B . Women admitted to secure forensic psychiatry services: I. Comparison of women and men. J Forensic Psychiatry. (2000) 11:275–95. doi: 10.1080/09585180050142525

[13] Bland J Mezey G Dolan B . Special women, special needs: A descriptive study of female special hospital patients. J Forensic Psychiatry. (1999) 10:34–45. doi: 10.1080/09585189908402137

[14] Mezey G Hassell Y Bartlett A . Safety of women in mixed-sex and single-sex medium secure units: staff and patient perceptions. Br J Psychiatry. (2005) 187:579–82. doi: 10.1192/bjp.187.6.579, PMID: 16319412

[15] Sarkar J di Lustro M . Evolution of secure services for women in England. Adv Psychiatr Treat. (2011) 17:323–31. doi: 10.1192/apt.bp.109.007773

[16] Tomlin J Lega I Braun P Kennedy HG Herrando VT Barroso R . ; experts of COST Action IS1302. Forensic mental health in Europe: some key figures. Soc Psychiatry Psychiatr Epidemiol. (2021) 56:109–17. doi: 10.1007/s00127-020-01909-6, PMID: 32651594 PMC7847441

[17] Rutherford M Duggan S . Forensic mental health services: facts and figures on current provision. Br J Forensic Practice. (2008) 10:4–10. doi: 10.1108/14636646200800020

[18] Harty M Somers N Bartlett A . Women's secure hospital services: national bed numbers and distribution. J Forensic Psychiatry Psychol. (2012) 23:590–600. doi: 10.1080/14789949.2012.717300, PMID: 23236263 PMC3516819

[19] Bartlett A Walker T Harty MA Abel KM . Health and social care services for women offenders: current provision and a future model of care. J Forensic Psychiatry Psychol. (2014) 25:625–35. doi: 10.1080/14789949.2014.944202

[20] NHS England . Implementing the five year forward view for mental health(2016). Available online at: https://www.england.nhs.uk/wp-content/uploads/2016/07/fyfv-mh.pdf (Accessed June 24, 2025).

[21] NHS England . NHS-Led Provider Collaboratives: specialised mental health, learning disability and autism services(2019). Available online at: https://www.england.nhs.uk/mental-health/nhs-led-provider-collaboratives/ (Accessed June 24, 2025).

[22] Department of Health . Review of health and social services for mentally ill offenders and others requiring similar services (The Reed Report). London: Department of Health (1992).

[23] Long C Hall L Craig L Mochty U Hollin CR . Women referred for medium secure inpatient care: a population study over a six-year period. J Psychiatr Intensive Care. (2011) 7:17–26. doi: 10.1017/S1742646410000099

[24] Kasmi Y Duggan C Völlm B . A comparison of long-term medium secure patients within NHS and private and charitable sector units in England. Criminal Behav Ment Health. (2020) 30:38–49. doi: 10.1002/cbm.2141, PMID: 32173951

[25] Maden A Skapinakis P Lewis GH Scott F Burnett R Jamieson E . Gender differences in reoffending after discharge from medium secure units: National cohort study in England and Wales. Br J Psychiatry. (2006) 189:168–72. doi: 10.1192/bjp.bp.105.014613, PMID: 16880488

[26] Ribeiro RB Tully J Fotiadou M . Clinical characteristics and outcomes on discharge of women admitted to a Medium Secure Unit over a 4-year period. Int J Law Psychiatry. (2015) 39:83–9. doi: 10.1016/j.ijlp.2015.01.025, PMID: 25748885

[27] Tully J Cappai A Lally J Fotiadou M . Follow-up study of 6.5 years of admissions to a UK female medium secure forensic psychiatry unit. BJPsych Bull. (2019) 43:54–7. doi: 10.1192/bjb.2018.77, PMID: 30257727 PMC6472308

[28] Milne S Barron P Fraser K Whitfield E . Sex differences in patients admitted to a regional secure unit. Medicine Sci Law. (1995) 35:57–60. doi: 10.1177/002580249503500112, PMID: 7877476

[29] Bartlett A Somers N Fiander M Harty MA . Pathways of care of women in secure hospitals: which women go where and why. Br J Psychiatry. (2014) 205:298–306. doi: 10.1192/bjp.bp.113.137547, PMID: 25104832

[30] Smith J Parker J Donovan M . Female admissions to a regional secure unit. J Forensic Psychiatry. (1991) 2:95–102. doi: 10.1080/09585189108408621

[31] Long CG Fitzgerald KA Hollin CR . Women firesetters admitted to secure psychiatric services: characteristics and treatment needs. Victims Offenders: Int J Evidence-Based Research Policy Practice. (2015) 10:341–53. doi: 10.1080/15564886.2014.967901

[32] Hollin CR Davies S Duggan C Huband N McCarthy L Clarke M . Patients with a history of arson admitted to medium security: characteristics on admission and follow-up postdischarge. Medicine Sci Law. (2013) 53:154. doi: 10.1258/msl.2012.012056, PMID: 23185072

[33] Adshead G . Damage: Trauma and violence in a sample of women referred to a forensic service. Behav Sci Law. (1994) 12:235–49. doi: 10.1002/bsl.2370120304

[34] Long CG Dolley O Barron R Hollin CR . Women transferred from prison to medium-secure psychiatric care: the therapeutic challenge. J Forensic Psychiatry Psychol. (2012) 23:261–73. doi: 10.1080/14789949.2012.674151

[35] Long CG Dolley O Hollin CR . Treatment progress in medium security hospital settings for women: changes in symptoms, personality and service need from admission to discharge. Criminal Behav Ment Health. (2015) 25:99–111. doi: 10.1002/cbm.1911, PMID: 24865814

[36] James K Stewart D Bowers L . Self-harm and attempted suicide within inpatient psychiatric services: a review of the literature. Int J Ment Health Nurs. (2012) 21:301–9. doi: 10.1111/j.1447-0349.2011.00794.x, PMID: 22340085

[37] Völlm BA Dolan MC . Self-harm among UK female prisoners: a cross-sectional study. J Forensic Psychiatry Psychol. (2009) 20:741–51. doi: 10.1080/14789940903174030

[38] Laporte N Klein TS Ozolins A Westrin Å Westling S Wallinius M . Emotion regulation and self-harm among forensic psychiatric patients. Front Psychol. (2021) 12:710751. doi: 10.3389/fpsyg.2021.710751, PMID: 34504461 PMC8421601

[39] Kaggwa MM Davids A Moulden H Chaimowitz GA Mohibi P Erb B . Self-harming behaviors among forensic psychiatric patients who committed violent offences: an exploratory study on the role of circumstances during the index offence and victim characteristics. BMC Psychiatry. (2025) 25:427. doi: 10.1186/s12888-025-06877-2, PMID: 40295960 PMC12039141

[40] Sarkar J . Short-term management of repeated self-harm in secure institutions. Adv Psychiatr Treat. (2011) 17:435–46. doi: 10.1192/apt.bp.110.008045

[41] Klonsky ED Moyer A . Childhood sexual abuse and non-suicidal self-injury: meta-analysis. Br J Psychiatry. (2008) 192:166–70. doi: 10.1192/bjp.bp.106.030650, PMID: 18310572

[42] Holden R Stables I Brown P Fotiadou M . Adverse childhood experiences and adult self-harm in a female forensic population. BJPsych Bull. (2022) 46:148–52. doi: 10.1192/bjb.2021.34, PMID: 33958018 PMC9347066

[43] Coid J Hickey N Kahtan N Zhang T Yang M . Patients discharged from medium secure forensic psychiatry services: reconvictions and risk factors. Br J Psychiatry. (2007) 190:223–9. doi: 10.1192/bjp.bp.105.018788, PMID: 17329742

[44] Sahota S Davies S Duggan C Clarke M Huband N Owen V . Women admitted to medium secure care: their admission characteristics and outcome as compared with men. Int J Forensic Ment Health. (2010) 9:110–7. doi: 10.1080/14999013.2010.499555

[45] Tolland H McKee T Cosgrove S Drysdale E Gillespie M Paterson L . A systematic review of effective therapeutic interventions and management strategies for challenging behaviour in women in forensic mental health settings. J Forensic Psychiatry Psychol. (2019) 30:570–93. doi: 10.1080/14789949.2019.1627387

[46] Westhead J Gibbon S McCarthy L Hatcher R Clarke M . Long-term outcomes after discharge from medium secure care: still a cause for concern? J Forensic Psychiatry Psychol. (2023) 34:166–78. doi: 10.1080/14789949.2023.2190535, PMID: 17602128

[47] Davies S Clarke M Hollin C Duggan C . Long-term outcomes after discharge from medium secure care: a cause for concern. Br J Psychiatry. (2007) 191:70–4. doi: 10.1192/bjp.bp.106.029215, PMID: 17602128

[48] National health service act(2006). Available online at: https://www.legislation.gov.uk/ukpga/2006/41/section/251 (Accessed November 25, 2025).

[49] Mental health act(1983). Available online at: https://www.legislation.gov.uk/ukpga/1983/20/section/127 (Accessed November 25, 2025).

[50] World Health Organization . The ICD-10 Classification of Mental and Behavioural Disorders. Genève, Switzerland: World Health Organization (1993).

